# Clinical, laboratory, and histopathological characteristics of pediatric lupus nephritis: a retrospective study in a national referral center in Mexico

**DOI:** 10.3389/fped.2026.1743610

**Published:** 2026-03-05

**Authors:** Héctor Menchaca-Aguayo, Abril Bernabe-Jiménez, Karla Chacón-Abril, Enrique Faugier-Fuentes

**Affiliations:** 1 Department of Pediatric Rheumatology, Hospital General Dr. Agustín O’Horán, Mérida, Yucatán, Mexico; 2 Department of Pediatric Rheumatology, Hospital Infantil de México Federico Gómez, Mexico, Mexico; 3 Division of Rheumatology, Instituto Nacional de Enfermedades Respiratorias, Mexico, Mexico

**Keywords:** hypoalbuminemia, lupus nephritis, pediatric systemic lupus erythematosus, prognostic biomarkers, renal replacement therapy

## Abstract

**Background:**

Pediatric lupus nephritis (LN) remains a major cause of morbidity and mortality, yet data from Latin American populations are limited. This study aimed to describe the clinical, laboratory, and histopathological characteristics of pediatric LN and identify prognostic factors associated with renal replacement therapy (RRT).

**Methods:**

We conducted a retrospective cross-sectional study including patients <18 years of age with LN diagnosed between 2020 and 2024 at a national referral center in Mexico. Demographic, clinical, immunological, histopathological, and therapeutic variables at diagnosis were analyzed. Multivariable logistic regression was performed to identify predictors of RRT.

**Results:**

Eighty patients were included (83% female; mean age 15.1 ± 2.8 years). Median proteinuria was 41 mg/m²/h; hematuria and leukocyturia were present in 46% and 26% of patients, respectively. All patients were ANA positive, with frequent hypocomplementemia and elevated anti–double-stranded DNA titers. Among biopsied patients, class IV was the most common histological subtype (60%). Proliferative forms were associated with reduced glomerular filtration rate (<90 mL/min/1.73 m²; *p* = 0.012) and higher activity index scores (*p* = 0.04), while chronicity indices were low. Fifteen patients (18.8%) required RRT, and mortality was 6.25%. In multivariable analysis, hypoalbuminemia (<2.5 g/dL) was independently associated with RRT (OR 6.04; 95% CI 1.33–27.50; *p* = 0.020).

**Conclusions:**

This study represents one of the largest pediatric LN cohorts reported from Mexico. Proliferative forms were associated with greater inflammatory activity and impaired renal function at diagnosis. Hypoalbuminemia emerged as a simple and accessible biomarker for early risk stratification of severe renal outcomes.

## Introduction

Systemic lupus erythematosus (SLE) is a multisystem autoimmune disease characterized by loss of immune tolerance and autoantibody production (1). In children, SLE accounts for approximately 10%–20% of all cases and is associated with a more aggressive disease course and higher rates of major organ involvement, particularly the kidneys (2). Lupus nephritis (LN) is one of the most severe manifestations of pediatric SLE, affecting up to 30%–50% of patients and representing a major contributor to morbidity and long-term renal damage (3). The diagnosis and classification of LN rely on clinical findings, serological markers, and histopathological evaluation through renal biopsy, according to the International Society of Nephrology/Renal Pathology Society (ISN/RPS) classification (4, 5). This classification remains essential for disease stratification and therapeutic decision-making. The diagnostic and therapeutic approach to LN in pediatric patients remains a clinical challenge. Despite advances in immunosuppressive therapy, pediatric LN continues to pose significant diagnostic and therapeutic challenges, particularly in settings with limited access to specialized care (6, 7). Previous studies have identified several clinical, laboratory, and histopathological factors—such as histological class, degree of proteinuria, hypoalbuminemia, and serological activity—as being associated with adverse renal outcomes, including progression to chronic kidney disease or the need for renal replacement therapy (RRT) (8, 9). However, most available data derive from Asian populations or large international cohorts, limiting their generalizability to Latin American settings. In Mexico, published data on pediatric LN remain limited, and comprehensive descriptions of clinical and histopathological patterns are scarce. Characterizing these features in local cohorts may support earlier risk stratification and inform clinical decision-making, particularly using widely available clinical and laboratory parameters. Therefore, the aim of this study was to describe the clinical, laboratory, and histopathological characteristics of pediatric LN in a Mexican referral center and to explore factors associated with the need for RRT.

## Materials and methods

A retrospective observational study with cross-sectional analysis at LN diagnosis was conducted at the Hospital Infantil de México Federico Gómez. Patients younger than 18 years of age with a confirmed diagnosis of SLE who developed LN were included. All patients fulfilled the 1997 American College of Rheumatology (ACR) or the 2012 Systemic Lupus International Collaborating Clinics (SLICC) classification criteria for SLE, and were treated between January 2020 and December 2024. Renal involvement was defined by the presence of proteinuria, hematuria, urinary casts, reduced GFR, or compatible histopathological findings attributable to SLE. Patients with SLE without renal involvement and those with incomplete medical records were excluded. Induction therapy was defined as the initial immunosuppressive strategy initiated at the time of LN diagnosis. In our institution, induction follows a standardized multitarget approach, consisting of high-dose corticosteroids and cyclophosphamide, with early initiation of mycophenolate mofetil as part of the same induction phase rather than as single-agent induction therapy. When renal biopsy was available, cyclophosphamide was reserved for patients with proliferative LN (ISN/RPS classes III, IV, and V, including mixed forms). In patients with class I or II disease, induction consisted of corticosteroids and mycophenolate mofetil. In cases in which renal biopsy could not be performed due to logistical limitations, parental refusal, or clinical contraindications, the same multitarget induction strategy was initiated based on clinical and laboratory features consistent with active LN. Corticosteroid dosing followed a standardized institutional regimen for childhood-onset SLE during the induction phase. The analysis was intentionally restricted to variables available at the time of LN diagnosis. Longitudinal treatment data, including cumulative glucocorticoid exposure or steroid dose at 1 or 2 years, were not systematically available due to the retrospective and cross-sectional study design, and therefore were not evaluated as study outcomes. Alternative immunosuppressive agents were selected in specific clinical scenarios. Azathioprine was used in selected cases with severe extrarenal manifestations, such as central nervous system involvement or systemic vasculitis, in combination with cyclophosphamide during induction, whereas methotrexate was preferentially used in patients with concomitant severe articular involvement requiring disease-modifying therapy. In selected cases, rituximab was used as an alternative induction agent instead of cyclophosphamide, particularly in patients with severe extrarenal manifestations such as refractory or severe arthritis or neuropsychiatric involvement. Intravenous immunoglobulin (IVIG) and therapeutic plasma exchange (TPE) were reserved for patients with severe or life-threatening presentations, including those with concomitant sepsis or critical systemic involvement, and were used as adjunctive therapies according to institutional practice. These treatment decisions were made according to predefined institutional practices and were not intended for comparative effectiveness analyses. The information analyzed corresponded to the time of LN diagnosis. Given the retrospective and cross-sectional design of the study, the analysis was limited to variables available at disease onset, and longitudinal outcomes were not evaluated. Renal biopsies were centrally reviewed by a single expert nephropathologist, based on the ISN/RPS classification. Demographic, clinical, immunological, and histopathological data were collected, including age at symptom onset and diagnosis, clinical manifestations, and laboratory findings. Antinuclear antibodies were determined by indirect immunofluorescence (IIF) on HEp-2 cells, with titers ≥1:80 considered positive. Renal involvement was assessed based on kidney biopsy findings, including the activity index (AI) and chronicity index (CIx), using a standardized data collection form. All laboratory variables corresponded to values obtained at LN diagnosis, prior to initiation of induction therapy. The study protocol was approved by the local ethics committee (approval number HIM/SR/2025/005). As this was a retrospective review of anonymized data, written informed consent was not required.

## Statistical analysis

Data were recorded in an electronic database. Quantitative variables were described as means with standard deviation (SD) or as medians with interquartile range (IQR), according to their distribution. Categorical variables were expressed as frequencies and percentages. For group comparisons, the Student's *t*-test was used for normally distributed quantitative variables, and the Mann–Whitney *U*-test for non-normally distributed variables. Categorical variables were compared using the chi-square test or Fisher's exact test, depending on the number of expected cells. Binary univariate and multivariable logistic regression analyses were performed to identify factors associated with the need for RRT. Given the limited number of RRT events, the multivariable model was intentionally parsimonious and included only clinically relevant variables to minimize overfitting. Odds ratios (OR) with corresponding 95% confidence intervals (95% CI) were reported. Statistical significance was set at *p* < 0.05. Data were analyzed using *Stata* version 18.0 (StataCorp, College Station, TX, USA).

## Results

**Figure 1:**
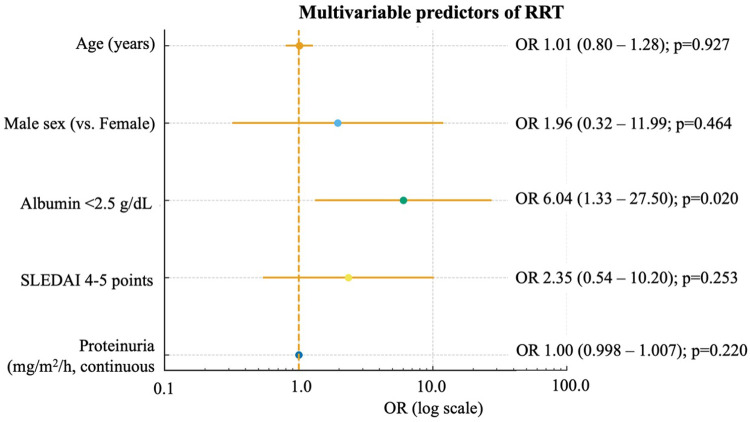
Forest plot showing multivariable logistic regression predictors of renal replacement therapy (RRT) in pediatric lupus nephritis (LN). The model was adjusted for age, sex, serum albumin level, SLEDAI score, and proteinuria (log scale). Odds ratios (OR) are shown with 95% confidence intervals (CI).

Two hundred medical records of patients with SLE were reviewed, of which 80 (40%) had LN and were included in the study. The majority were female (83%), with a mean age of 15.1 ± 2.8 years. The median time from symptom onset to diagnosis was 3 months. Fifteen patients (18.8%) required RRT at, or shortly after, LN presentation, and overall mortality was 6.25%. Of the five patients who died, one died at the time of diagnosis and four later due to hospital complications. The causes of death were septic shock (*n* = 3), ischemic stroke (*n* = 1), and hemorrhagic stroke (*n* = 1). In laboratory studies, more than one-third of the patients (38.75%) showed some degree of GFR impairment, predominantly mild (GFR 60–89 mL/min/1.73 m²) (64.5%). In urinalysis, 46% had hematuria, 35% had casts, and 26% leukocyturia. Median 12-hour proteinuria was 41 mg/m²/h. In the autoimmune profile, all patients were ANA positive, and most presented high anti-dsDNA titers. Hypocomplementemia (defined as low C3 and/or C4 levels) was observed in 90% of patients, with low C3 in 88.75% and low C4 in 77.5%. Among the specific autoantibodies, the most prevalent were anti-nucleosome (80%), and anti-RNP (41%) and anti-Sm (23%). Antiphospholipid antibodies (aPL) were frequently detected. Specifically, lupus anticoagulant was present in 42% of patients, followed by anti-β2 glycoprotein I IgM (31.6%) and anticardiolipin IgM (12.8%) (Table 1). Multiple aPL positivity was not associated with an increased requirement for RRT; none of the patients with multiple aPL positivity required dialysis, compared with 15 of 62 patients without multiple positivity (0/18 vs. 15/62; Fisher's exact test, *p* = 0.018). Of the 80 patients included, the Direct Antiglobulin Test (DAT) was performed in 37, of whom 34 (91.9%) had a positive result. Regarding clinical manifestations, 70% of patients showed extrarenal involvement, predominantly hematological (81%), followed by arthritis (56.3%), mucocutaneous involvement (54%), and neuropsychiatric manifestations (34%). Initial treatment included methylprednisolone pulses in 91% and cyclophosphamide in 78%. In refractory cases, rituximab (12.5%), IVIG (2.5%), or TPE (2.5%) were used. For maintenance therapy, mycophenolate mofetil was most frequent (61%), followed by azathioprine (30%). All patients received hydroxychloroquine and calcium/vitamin D supplementation. A total of 67.5% were treated with angiotensin-converting enzyme inhibitors (ACEIs) or angiotensin II receptor blockers (ARBs). Treatments are summarized in Table 2. These treatment patterns reflect the application of institutional treatment protocols as described in the Materials and Methods section. Forty-five biopsies were performed, with class IV being the most frequent (60%), followed by combinations III + V and IV + V. Overall, proliferative forms accounted for more than 80% of cases. Therefore, histopathological proportions refer exclusively to the subset of patients who underwent renal biopsy. The GFR showed a significant difference among histological classes (*p* = 0.012). The proportion of patients with GFR < 90 mL/min/1.73 m² increased markedly in classes III + V, IV, and IV + V, whereas classes I, II, and II + V maintained normal renal function. The activity index (AI) increased progressively with histological severity (medians: class III ≈ 5, class III + V ≈ 9.5, and class IV ≈ 12; *p* = 0.04), with higher median values in proliferative classes. In contrast, the chronicity index (CIx) was low (median = 1) and showed no significant differences among classes (*p* = 0.91). Comparisons between histological classes are shown in Table 3, highlighting that hypoalbuminemia (<2.5 g/dL) was significantly more frequent in class IV and mixed classes (*p* = 0.009), and that leukocyturia showed significant differences between groups (*p* = 0.013). Likewise, a comparative analysis was performed between patients with and without the need for RRT (Supplementary Table 1). Significant differences were identified in renal function at diagnosis, serum albumin levels, and proteinuria between patients who did and did not require RRT. In multivariable analysis, hypoalbuminemia emerged as the only clinical factor independently associated with the requirement of RRT (OR 6.04; 95% CI: 1.33–27.50; *p* = 0.020), as shown in Figure 1 and Supplementary Figure 1, Table 2.

**Table 1:** Clinical, laboratory, and histopathological characteristics of pediatric patients with lupus nephritis (LN) (*n* = 80; 45 renal biopsies classified according to the ISN/RPS system).

Variable	Result
Sociodemographic	
Age (years), mean ± SD	15.1 ± 2.8
Female, *n* (%)	67 (83.8)
Time from symptom onset to diagnosis, median (IQR)	3 months (1–9)
Renal function	
Serum creatinine (mg/dL), median (IQR)	0.63 (0.48–0.91)
GFR (mL/min/1.73 m²), mean ± SD	105.4 ± 42.7
GFR <90 mL/min/1.73 m², *n* (%)	31 (38.8)
Serum albumin (g/dL), mean ± SD	2.72 ± 0.94
12-h proteinuria (mg/m²/h), median (IQR)	41 (25–118.5)
Hematuria (>5 RBCs/field), *n* (%)	37 (46.3)
Leukocyturia (>5 WBCs/field), *n* (%)	21 (26.3)
Casts, *n* (%)	28 (35.0)
Quantitative autoimmunity	
ANA	100% positive
Anti-dsDNA (IU/mL), median (IQR)	584.5 (149.5–800)
C3 (mg/dL), median (IQR)	37.1 (23.5–60.8)
C4 (mg/dL), median (IQR)	4.65 (2.4–9.1)
Qualitative autoimmunity, *n*/*N* (%)	
Anti-Sm	16/68 (23.5)
Anti-RNP	12/29 (41.4)
Anti-Ro	2/8 (25.0)
Anti-La	2/8 (25.0)
Anti-nucleosome	56/70 (80.0)
Lupus anticoagulant	32/77 (41.6)
Anticardiolipin IgG	7/79 (8.9)
Anticardiolipin IgM	10/78 (12.8)
Anti-β2 glycoprotein I IgG	4/79 (5.1)
Anti-β2 glycoprotein I IgM	25/79 (31.6)
Clinical manifestations	
Hematologic, *n* (%)	65 (81.3)
Arthritis, *n* (%)	45 (56.3)
Mucocutaneous, *n* (%)	43 (53.8)
Neuropsychiatric, *n* (%)	27 (33.8)
Renal histopathology (ISN/RPS classification)	
Class I	1 (2.2)
Class II	3 (6.7)
Class III	4 (8.9)
Class IV	27 (60.0)
Class V	2 (4.4)
Mixed classes (III + V, IV + V, II + V)	8 (17.8)
Activity index (AI), median (IQR)	8 (6–13)
Chronicity index (CI), median (IQR)	1 (0–2)

Data are presented as mean ± SD, median (IQR), or frequency (%). AI, activity index; CI, chronicity index; ANA, antinuclear antibodies; GFR, glomerular filtration rate.

**Table 2:** Initial, subsequent, and supportive treatments, and frequency of renal replacement therapy (RRT) and mortality.

Variable	*n* (%)
Initial treatment	
Intravenous methylprednisolone (pulses)	73 (91.3)
Cyclophosphamide	62 (77.5)
Rituximab	10 (12.5)
Intravenous immunoglobulin (IVIG)	2 (2.5)
Therapeutic plasma exchange (TPE)	2 (2.5)
Subsequent treatment	
Hydroxychloroquine	80 (100.0)
Mycophenolate mofetil (MMF)	49 (61.3)
Azathioprine	24 (30.0)
Methotrexate	7 (8.8)
Supportive/preventive treatment	
Antihypertensives (ACEIs, ARBs)	54 (67.5)
Calcium and vitamin D	80 (100.0)
Anticoagulation	14 (17.5)
Outcomes	
Renal replacement therapy (RRT)	15 (18.8)
Mortality	5 (6.3)

Summary of initial, subsequent, and supportive treatments administered to pediatric patients with lupus nephritis, and frequency of renal replacement therapy (RRT) and mortality. Data are expressed as *n* (%). ACEIs, angiotensin-converting enzyme inhibitors; ARBs, angiotensin II receptor blockers; IVIG, intravenous immunoglobulin; TPE, therapeutic plasma exchange; MMF, mycophenolate mofetil; RRT, renal replacement therapy.

**Table 3:** Clinical and laboratory comparisons across lupus nephritis (LN) classes.

Variable	Key finding	*p*-value
SLEDAI (high/very high activity, %)	Increased in proliferative classes (III + V, IV, IV + V)	0.08
GFR <90 mL/min/1.73 m² (%)	Decreased mainly in proliferative classes (III + V, IV, IV + V)	**0.012**
Albumin <2.5 g/dL (%)	81.5% in class IV and combined classes	**0.009**
12-h proteinuria (mg/m²/h)	Higher in proliferative classes (IV, IV + V, V)	0.14
Hematuria (%)	Present in 37% of class IV and 100% of class V cases	0.17
Leukocyturia (%)	55.6% in class IV; significant differences among classes	**0.013**
Casts (%)	51.9% in class IV; variable across other classes	0.98
Hypertension at diagnosis (%)	Common in class IV (40.7%) and present in 100% of classes I and II	0.11
Activity index (AI), median (IQR)	Increased with histological severity; highest in class IV and combined classes	**0.04**
Chronicity index (CIx), median (IQR)	Low overall, without significant differences between classes	0.91

Proliferative classes (III, III + V, IV) showed higher AI compared with non-proliferative forms. Bold values indicate statistical significance (*p* < 0.05). SLEDAI, systemic lupus erythematosus disease activity index; GFR, glomerular filtration rate; AI, activity index; CIx, chronicity index.

## Discussion

This study describes the clinical, laboratory, and histopathological characteristics of a Mexican cohort of pediatric patients with LN diagnosed at a national referral center. Consistent with previous pediatric series, a clear female predominance and presentation during adolescence were observed (10). Notably, the median time from symptom onset to diagnosis was longer than that reported in other cohorts (8), which may reflect diagnostic delays and heterogeneity in initial presentation. At diagnosis, a substantial proportion of patients already exhibited impaired renal function and markers of active glomerular disease, underscoring the high burden of renal severity at presentation, particularly in patients with proliferative forms of LN (11, 12). From an immunological perspective, all patients were ANA positive, with the highest titers corresponding to anti-dsDNA and anti-nucleosome antibodies, both consistently associated with increased disease activity and severe renal involvement (13). Hypocomplementemia was also frequent, as expected due to complement consumption in active LN (14). aPL positivity was frequently observed, particularly in patients with severe disease, potentially contributing to greater renal severity through mechanisms related to vascular injury and microthrombosis (15). Interestingly, multiple aPL positivity was not associated with an increased need for RRT in our cohort, and no patient with double or triple aPL positivity required dialysis, suggesting that aPL burden alone may not be sufficient to predict severe renal outcomes in pediatric LN. Direct antiglobulin test (DAT) positivity was observed more frequently than typically reported in pediatric SLE cohorts (16). In our cohort, this elevated frequency likely reflects the high burden of hematological involvement and a more active immunological profile at diagnosis. However, DAT positivity was not a predefined outcome of this study and should be interpreted as an exploratory secondary finding, without direct implications for renal severity or outcomes. Renal biopsies confirmed a predominance of proliferative forms, with class IV being the most frequent (60%). These findings are consistent with international pediatric cohorts, where classes III and IV represent the majority of cases (7). The median histological AI was 8, reflecting marked renal inflammation at diagnosis, while the CIx was low, suggesting potential reversibility with intensive immunosuppressive therapy (17). In our cohort, the AI increased proportionally with histological severity (class III ≈ 5; III + V ≈ 9.5; IV ≈ 12; *p* = 0.04), indicating a higher burden of active lesions (endocapillary proliferation, necrosis, and crescents) in proliferative classes. This pattern is consistent with recent reports showing that class IV (±V) exhibits the highest AI values, while classes I/II show near-zero AI (18). Moreover, no relevant differences were observed in the distribution of the CIx among classes in initial cross-sectional evaluations. Current international guidelines recommend kidney biopsy in all patients with suspected LN to guide therapeutic decisions and assess prognosis. In our cohort, renal biopsy could not be performed in all patients, reflecting routine clinical practice in a high-volume public referral center within a resource-limited setting. Factors such as limited procedural availability, high patient turnover, parental refusal, and clinical contraindications at presentation contributed to this finding. In selected cases with a clear clinical and laboratory presentation of active LN who showed early response to induction therapy, renal biopsy was deferred, prioritizing biopsy resources for patients in whom histological information was expected to have a direct impact on therapeutic decision-making. Therefore, histopathological analyses should be interpreted within the context of patients who underwent renal biopsy, rather than the entire cohort. These findings highlight the challenges of fully implementing guideline-recommended care in routine practice and underscore the importance of contextualizing biopsy rates when interpreting observational data. The predominance of proliferative histological forms (mainly class IV) highlights the aggressive renal involvement observed in this cohort. These histopathological findings underscore the need for early and intensive immunosuppressive management at diagnosis in proliferative forms, in accordance with current international guidelines. Differences in histological severity across pediatric LN cohorts may be partially explained by ethnic and regional factors (a comparative overview of representative pediatric LN cohorts from different regions is provided in Supplementary Table 3). Prior studies have shown that early-onset SLE is more common and aggressive in Hispanic, African-American, and Asian populations compared with White patients (3). In this context, the high proportion of proliferative LN observed in our cohort likely reflects the combined influence of ethnicity, referral bias, and healthcare access at diagnosis. In contrast, several cohorts from low- and middle-income regions, including Latin America, have described a higher burden of disease severity at presentation and less favorable renal outcomes, although with considerable heterogeneity across regions and healthcare systems (19). These differences highlight the importance of reporting region-specific data to better understand the global heterogeneity of pediatric LN and the contextual factors influencing disease severity. Initial management was characterized by extensive use of intravenous methylprednisolone pulses (91.25%) and cyclophosphamide (77.5%), reflecting the clinical severity at admission and the institutional preference for intensive regimens in multisystem disease. This therapeutic approach is consistent with international recommendations for severe LN presentations (20–22). In refractory or severe cases, rituximab (12.5%), IVIG (2.5%), and TPE (2.5%) were used, particularly in patients with severe extrarenal involvement, including neuropsychiatric manifestations, consistent with previous reports in refractory LN, catastrophic antiphospholipid syndrome, or hemophagocytic syndrome (23–25). For subsequent therapy, mycophenolate mofetil was the most frequently used drug (61.25%), followed by azathioprine (30%), reflecting a trend toward regimens with proven efficacy in relapse prevention (20). All patients received hydroxychloroquine, in line with international guidelines (20, 24), and all were prescribed calcium and vitamin D (100%) as preventive measures against glucocorticoid-associated bone demineralization (26). The use of ACE inhibitors/ARBs (67.5%) was consistent with the presence of proteinuria or hypertension (27), while anticoagulation (17.5%) was restricted to patients with antiphospholipid syndrome or high thrombotic risk (28). In terms of outcomes, 18.8% of patients required RRT, reflecting the high burden of severe disease in this cohort. This proportion is higher than reported in international consortia, where RRT rates range between 8% and 15% during early follow-up, although with variability according to disease severity and access to intensive immunosuppressive therapy (29–31). The observed mortality (6.25%) was lower than in historical series (up to 10%–15%), but still significant compared with recent cohorts from Asia and North America reporting rates around 2%–5% (9, 31). These differences may be related to the higher severity profile of cases in a tertiary referral center and to potential diagnostic delays. Clinical activity, assessed using the Systemic Lupus Erythematosus Disease Activity Index (SLEDAI), tended to be higher in proliferative forms, with 75%–100% of patients showing high or very high activity, compared with no high activity in classes I, II, and II + V (*p* = 0.08), consistent with the more aggressive course of these variants (32). Hypoalbuminemia was more frequent in class IV and combined classes (81.48%), with statistical significance (*p* = 0.009), underscoring its value as an indirect marker of renal severity (33). In our cohort, the association of reduced GFR at diagnosis, hypoalbuminemia, and higher magnitude of proteinuria in patients requiring RRT is consistent with evidence that proteinuria is the primary clinical prognostic marker in LN and that baseline renal dysfunction predicts adverse outcomes, especially in proliferative forms (34). In multivariate analysis, hypoalbuminemia was the only clinical factor significantly associated with RRT (OR 6.04; 95% CI: 1.33–27.50; *p* = 0.020), reinforcing its role as an adverse predictor of renal outcomes (35). This finding is clinically meaningful, as hypoalbuminemia likely reflects both urinary protein loss due to glomerular injury and a systemic inflammatory state characterized by increased capillary permeability. Importantly, unlike other more sophisticated and costly predictors—such as urinary biomarkers or advanced imaging techniques—serum albumin represents a simple, accessible, and cost-effective clinical biomarker, available in virtually any hospital setting (35). Its use in early risk stratification may help promptly identify patients at higher risk of renal failure, allowing for more intensive interventions and closer follow-up. Conversely, leukocyturia varied among histological classes (*p* = 0.013) but was not consistently associated with glomerular activity, suggesting its limited value as an isolated marker (36, 37). The limitations of this study are primarily related to its retrospective and cross-sectional design, which restricted the analysis to variables available at the time of LN diagnosis and precluded evaluation of longitudinal outcomes, including treatment response, relapse rates, and long-term renal evolution. The lack of standardized longitudinal data on glucocorticoid exposure beyond the induction phase represents an important limitation of this study and precludes evaluation of steroid dosing at later follow-up time points. In addition, social and geographic factors such as distance from the referral center, time to referral, and access to specialized care were not systematically collected or analyzed, which may have influenced disease severity at presentation. As a national public referral center, our institution receives patients from diverse regions of the country, predominantly from central Mexico, with wide variability in geographic distance and access to specialized care. These factors may contribute to delays in referral and more severe disease at presentation, although they were not formally measured in this study. Moreover, the predominantly Latin American mestizo population included in this cohort may have distinct genetic and socioeconomic characteristics that could influence LN presentation and severity, an aspect that warrants further investigation. Furthermore, being conducted in a single tertiary referral center may have introduced selection bias by concentrating more severe cases, and the relatively small sample size limits generalizability and statistical power. Nevertheless, this study constitutes one of the largest reports in an exclusively Mexican pediatric population, providing valuable information from a Latin American reference center. Future multicenter and prospective studies with greater ethnic diversity are needed to confirm these findings, validate prognostic markers, and explore dynamic biomarkers of disease activity and therapeutic response.

## Conclusions

This study represents one of the largest pediatric LN cohorts reported from a Latin American referral center and the first focused exclusively on a Mexican population. Serum albumin levels <2.5 g/dL emerged as a simple, accessible, and independent predictor of the need for RRT, highlighting the prognostic value of early markers of renal damage at diagnosis. Although mortality was lower than that reported in historical series, it remained clinically significant compared with contemporary pediatric cohorts. Based on our experience, early identification of high-risk patients, timely referral to specialized centers, prompt initiation of intensive immunosuppressive therapy, and proactive management of complications such as infection and renal failure may contribute to improved survival in children with lupus nephritis.

## Data Availability

The original contributions presented in the study are included in the article/Supplementary Material, further inquiries can be directed to the corresponding author.
