# Supplementary material for: Clinical, laboratory, and histopathological characteristics of pediatric lupus nephritis: a retrospective study in a national referral center in Mexico
Source: Front Pediatr. 2026 Mar 5;14:1743610. doi: 10.3389/fped.2026.1743610 (PMC12999860; doi:10.3389/fped.2026.1743610)
Supplement: Supplementary file 1 [file Supplementaryfile1.docx]

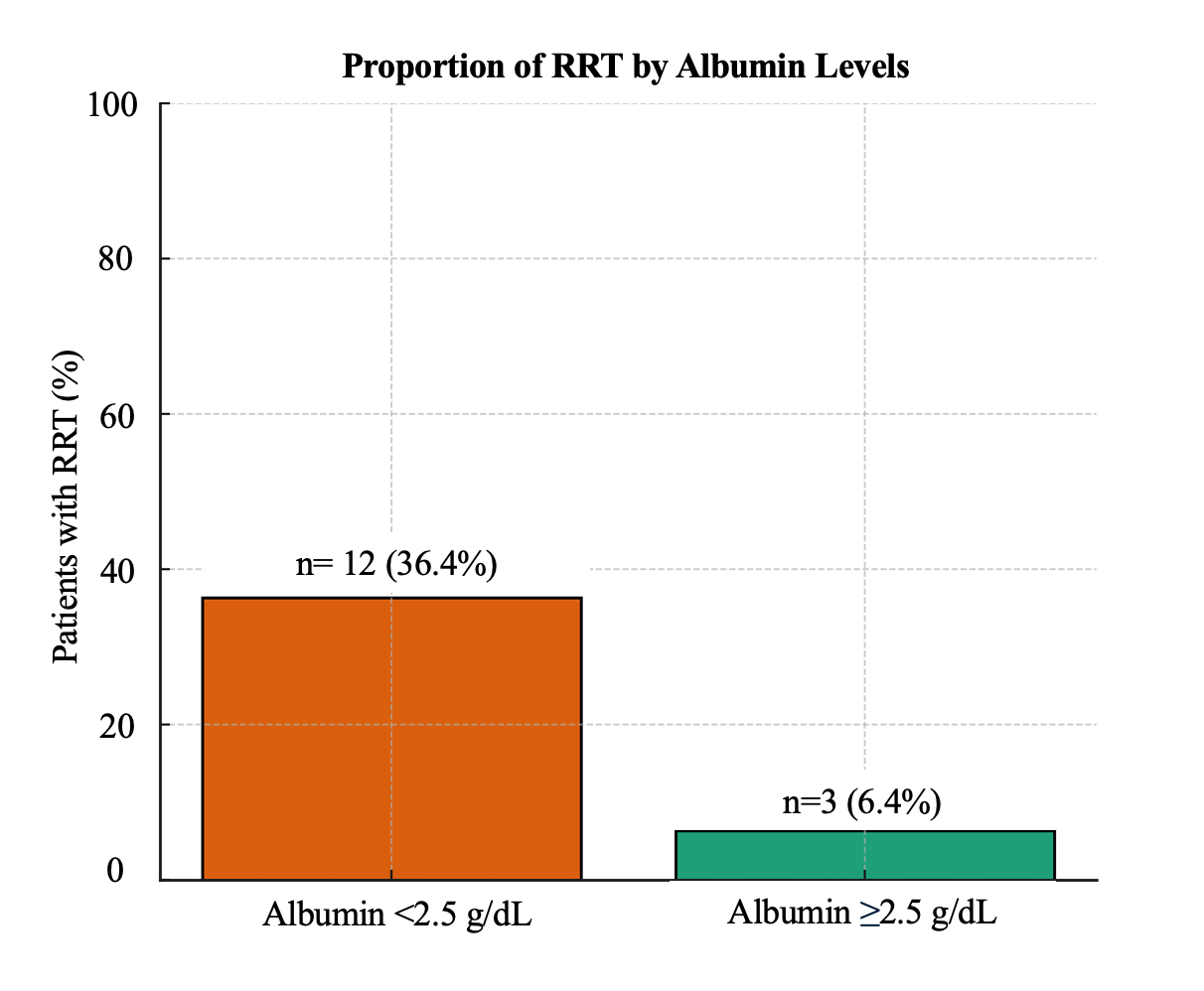
**Supplementary Fig. 1**. Bar chart showing the proportion of pediatric lupus nephritis (LN) patients requiring renal replacement therapy (RRT) according to serum albumin levels. Patients with hypoalbuminemia (<2.5 g/dL) had a significantly higher frequency of RRT (36.4%) compared to those with albumin ≥2.5 g/dL (6.4%) (OR 6.04; 95% CI: 1.33–27.50; p = 0.020).

**Supplementary Table 1**. Comparison of clinical, histological, and laboratory characteristics between patients with and without renal replacement therapy (RRT) requirement.

| **Variable** | **No RRT (n = 65)** | **RRT (n = 15)** | **p-value** |
| --- | --- | --- | --- |
| Hypertension at onset, n (%) | 36 (55.4) | 4 (26.7) | **0.04** |
| GFR <90 mL/min/1.73 m², n (%) | 19 (29.2) | 12 (80.0) | **0.001** |
| Albumin <2.5 g/dL, n (%) | 21 (32.3) | 12 (80.0) | **0.001** |
| 12-h proteinuria (mg/m²/h), median (IQR) | 38 (14–97) | 108 (77–292) | **0.0006** |
| Nephrotic-range proteinuria (>40 mg/m²/h), n (%) | 31 (47.7) | 14 (93.3) | **0.001** |
| Hematuria, n (%) | 39 (60.0) | 4 (26.7) | **0.02** |
| Leukocyturia, n (%) | 52 (80.0) | 7 (46.7) | **0.013** |
| Urinary casts, n (%) | 46 (70.8) | 6 (40.0) | **0.02** |

Data are presented as n (%) or median (interquartile range, IQR), as appropriate. Bold values indicate statistical significance (p < 0.05). GFR: glomerular filtration rate; RRT: renal replacement therapy.

**Supplementary Table 2**. Multivariable logistic regression model for predictors of renal replacement therapy (RRT).

Multivariable logistic regression analysis (n = 80; Pseudo R² = 0.2063; global p = 0.007).

| **Variable** | **OR (odds ratio)** | **95% CI** | **p-value** |
| --- | --- | --- | --- |
| Age (years) | 1.01 | 0.80–1.28 | 0.927 |
| Male sex (vs female) | 1.96 | 0.32–11.99 | 0.464 |
| Albumin <2.5 g/dL | **6.04** | **1.33–27.50** | **0.020** |
| SLEDAI 4–5 points | 2.35 | 0.54–10.20 | 0.253 |
| Proteinuria (mg/m²/h, continuous) | 1.00 | 0.998–1.007 | 0.220 |
| Constant (_cons) | 0.00054 | 9.94e–07–0.2938 | **0.019** |

Binary logistic regression was used to estimate the association between clinical variables and the outcome of renal replacement therapy. Bold values indicate statistical significance (p < 0.05). CI: confidence interval; OR: odds ratio; RRT: renal replacement therapy.

**Supplementary Table 3**. International comparison of pediatric lupus nephritis cohorts.

| **Study** | **Region** | **Cohort size (patients) / renal biopsies, n** | **Female (%)** | **Class IV (%)** | **Proliferative LN (%)** | **RRT / CKD stage 3–4 at LN presentation (%)** | **Mortality (%)** |
| --- | --- | --- | --- | --- | --- | --- | --- |
| Smitherman et al., 2023 | North America (USA, Canada) | 222 | 83% (184/222) | 36% (79/222) | 64% | Not reported | Not reported |
| Moral-Larraz et al., 2021 | Europe (Spain) | 15* | 68.8% (11/16) | 33.3% (5/15) | 80% | 0% | 0% |
| Khatri et al., 2025 | South Asia (Pakistan) | 25 | 80% (20/25) | 36% | 92% | 4% (CKD stage 3 at presentation) | 8% |
| Chan et al., 2023 | East Asia (Hong Kong) | 92 | 84.8% | 46.7% | 90.2% | 7.6% (RRT at diagnosis) | 2.2% |
| Asis et al., 2025 | Southeast Asia (Philippines) | 21† | 93% | 19% | 57% | Not reported | 19.5% |
| Park et al., 2025 | East Asia (South Korea) | 216 | 72.7% | 63.9% | 79.6% | Not reported | 2.8% |
| Biswas et al., 2023 | South Asia (Eastern India) | 60 | 80% | 57% (±V) | 85% | 0% (no RIFLE-F/P) | 17% |
| Sakamoto et al., 2023 | Latin America (Brazil) | 464‡ | 84.8% | Not reported | 56.9% | Not reported | 4.1% |
| Demir et al., 2021 | Europe (Turkey) | 53 | ~73% | 54.7% | 77.4% | 13.2% (CKD stages 3–5) | 5.7% |
| **Menchaca-Aguayo et al., 2026** | **Latin America (Mexico)** | **80 / 45** | **83%** | **60%** | **84.4%** | **18.8% (RRT at presentation)** | **6.25%** |

**Footnotes**

* Although 16 patients were included, only 15 underwent renal biopsy.

† Renal biopsy performed in a subset of the total cohort (21/128 patients).

‡ Renal biopsy performed in a subset of a larger cohort (464/1528 patients).

Histopathological proportions (Class IV and proliferative LN) are calculated exclusively among patients who underwent renal biopsy.

Renal outcomes correspond to findings at the time of lupus nephritis presentation unless otherwise specified.
